# Geospatial distribution of severe paediatric intussusception in KwaZulu-Natal province, South Africa

**DOI:** 10.11604/pamj.2020.36.320.19814

**Published:** 2020-08-21

**Authors:** Yoshan Moodley, Vineshree Mischka Moodley, Sitheni Samson Mashele, Ravi Pokala Kiran, Thandinkosi Enos Madiba

**Affiliations:** 1 Faculty of Health and Environmental Sciences, Central University of Technology, Bloemfontein, South Africa,; 2 Private Practice, Cape Town, South Africa,; 3 Columbia University Medical Center and Mailman School of Public Health, New York, USA,; 4 Department of Surgery, University of KwaZulu-Natal, Durban, South Africa

**Keywords:** Intussusception, paediatric, geographic mapping

## Abstract

**Introduction:**

intussusception in South African (SA) children is often severe. A proportion of cases require management at quaternary hospitals which are a scare resource in SA. A geospatial investigation of severe paediatric intussusception (SPI) in the KwaZulu-Natal (KZN) province of SA would assist with identifying regions which should be targeted for preventative interventions. This could reduce resource utilisation for this condition at quaternary hospitals. The objective of this study was to determine the geospatial distribution of SPI in KZN.

**Methods:**

this was a retrospective analysis of data for patients with SPI who were admitted to a quaternary hospital in KZN over an 11-year period. Data related to patient demographics, duration of hospitalization, surgical intervention, inpatient mortality and residential postal code were extracted from the electronic hospital admissions system. Each residential postal code was linked to a corresponding KZN district municipality. Descriptive statistical methods were used to determine the distribution of various characteristics in the study sample. Semi-quantitative geospatial analysis was used to determine the distribution of patients with SPI in each KZN district municipality.

**Results:**

the study sample consisted of 182 patients with SPI. Most patients were <1 year old (83.5%), male (51.1%) and black African (87.9%). All patients underwent surgical intervention. Inpatient mortality was 2.7%. The majority of patients in the study sample resided in the eThekwini and King Cetshwayo district municipalities (51.1% and 14.8%, respectively).

**Conclusion:**

preventative interventions for SPI should be considered for rollout in the eThekwini and King Cetshwayo district municipalities of KZN, SA.

## Introduction

Intussusception refers to the invagination of a proximal segment of the gastrointestinal tract within the lumen of the adjacent segment [1]. The condition may involve the ileum, the colon or both [2]. Although intussusception occurs in both adults and children, children appear to be disproportionately affected by the condition and it remains an important cause of intestinal obstruction in this group. The aetiology of paediatric intussusception is usually idiopathic, but a proportion of cases are caused by gastrointestinal infection [1]. Therefore, a proportion of paediatric intussusception cases, particularly those related to gastrointestinal infection, are potentially avoidable. The incidence of paediatric intussusception in South Africa (SA) has been estimated at 32 cases per 100 000 children, with the burden of disease being highest amongst the black African ethnic group [3]. A mortality rate of 9.4% has been reported in paediatric patients who have not received timely medical or surgical intervention for intussusception [3]. Conservative approaches, such as pneumatic reduction, are effective in managing paediatric intussusception in high-income countries [4], however success rates in SA settings are less convincing and surgery at specialist paediatric surgical units is often indicated [2,3]. Surgical intervention rates of up to 80% have been reported in SA settings [3]. This might be due to differences in the severity of paediatric intussusception between SA populations and populations in high-income settings [3]. A geospatial study of severe paediatric intussusception (SPI) in the KwaZulu-Natal (KZN) province of SA would have implications for paediatric health in this setting. Geographic locales identified as having a high number of patients with SPI can be targeted for public health interventions aimed at reducing the disease burden of SPI. This would have a subsequent impact on SPI-related healthcare resource utilization and healthcare expenditure within the specialized paediatric units of public-sector quaternary hospitals, which are a scare resource in SA. Therefore, the objective of this study was to perform a geospatial investigation of SPI in KZN, SA.

## Methods

**Study design:** this was a retrospective analysis of data for patients with SPI who were admitted to the Inkosi Albert Luthuli Central Hospital (IALCH) in the eThekwini municipality, KZN province, SA.

**Study setting:** the province of KZN is comprised of 11 district municipalities: Amajuba, eThekwini, Harry Gwala, iLembe, King Cetshwayo, Ugu, uMgungundlovu, uMkhanyakude, uMzinyathi, uThukela and Zululand. IALCH is the only quaternary-level, public-sector hospital in KZN. The healthcare services offered by IALCH are highly specialised and admission to the hospital is strictly referral-based. Most patients admitted to IALCH are referred from lower-level regional hospitals within KZN.

**Study sample:** patients admitted to IALCH are complex cases which cannot be successfully managed at lower-level healthcare facilities. Therefore, all patients admitted for the management of intussusception at this hospital were classified as having SPI. Eligible patients were identified following a review of the electronic hospital admissions system using the following search limits: age <18 years old; admission between the 1^st^ January 2008 and 31^st^ December 2018; International Classification of Diseases 10^th^ Revision (ICD-10) primary diagnosis code of K56.1.

**Data collection:** data related to each patients´ age, gender, ethnicity, duration of hospitalisation, whether surgery was performed, inpatient mortality, and residential postal code were extracted directly from the electronic hospital admissions system and retained as a Microsoft Excel® spreadsheet for subsequent data analysis. A publicly-available registry, maintained by the South African Post Office, was used to link each residential postal code to its corresponding KZN district municipality.

**Data analysis:** descriptive statistical methods were used to determine the distribution of various characteristics in the study sample, with the results being presented as frequencies and percentages or medians with interquartile range (IQR). The descriptive statistical analysis was performed using the Statistical Package for the Social Sciences version 25.0 (IBM Corp, USA). Semi-quantitative geospatial analysis was used to determine the distribution of patients with SPI in KZN. Briefly, the residential postal address for each patient in the study sample and the Power Map® add-in software for Microsoft Excel® was used to map SPI according to KZN district municipality. District municipalities which contributed patients toward the study sample were allocated a red dot on the map. The size of the red dot on the map was directly proportional to the number of patients with SPI originating from that specific district municipality.

**Ethical approval:** this research was approved by the Biomedical Research Ethics Committee of the University of KZN (Protocol: BE595/16).

## Results

**Figure 1:**
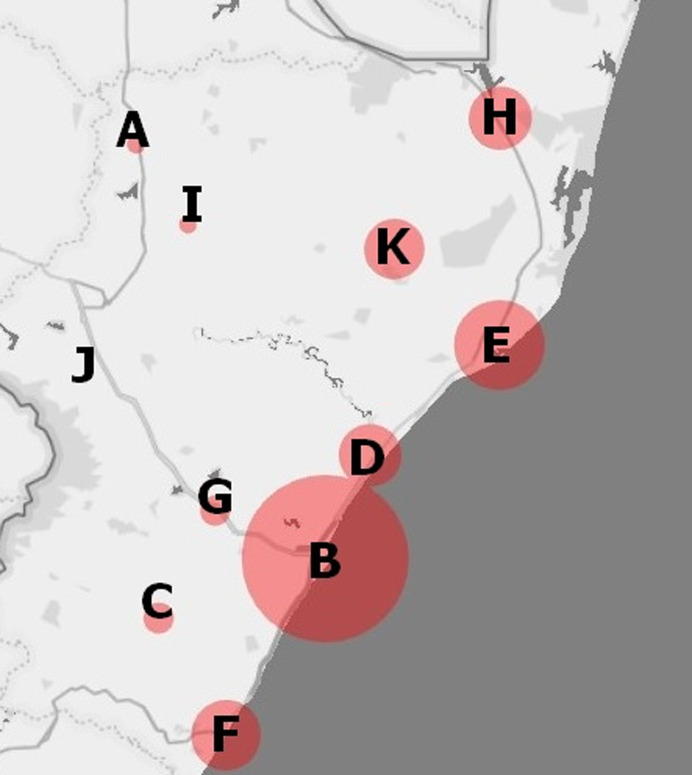
geospatial distribution of patients with SPI (N=182) in KZN by district municipality, 2008-2018

A review of the electronic hospital admissions system revealed that there were 182 patients, hereafter referred to as the “study sample”, admitted for the management of paediatric intussusception at IALCH between the 1^st^ January 2008 and 31^st^ December 2018. A description of the study sample is provided in Table 1. The geospatial distribution of patients with SPI in KZN is presented in Figure 1. The distribution of the study sample by district municipality was as follows: Amajuba - A: 1 (0.5%), eThekwini - B: 93 (51.1%), Harry Gwala - C: 3 (1.6%), iLembe - D: 13 (7.1%), King Cetshwayo - E: 27 (14.8%), Ugu - F: 16 (8.8%), uMgungundlovu - G: 3 (1.6%), uMkhanyakude - H: 13 (7.1%), uMzinyathi - I: 1 (0.5%), uThukela - J: 0 (0.0%) and Zululand - K: 12 (6.6%).

**Table 1:** description of the study sample (N=182)

Characteristic	n (% N)
**Age**	
<1 year old	152 (83.5)
1-5 years old	28 (15.4)
>5 years old	2 (1.1)
**Gender**	
Male	93 (51.1)
Female	89 (48.9)
**Ethnicity**	
Black African	160 (87.9)
Indian	7 (3.8)
White	1 (0.5)
Other/unknown	14 (7.6)
**Season of admission**	
Spring (September-November)	51 (28.0)
Summer (December-February)	48 (26.4)
Autumn (March-May)	42 (23.1)
Winter (June-August)	41 (22.5)
**Duration of hospitalisation, days (IQR)**	5.0 (3.0-8.0)
**Surgical intervention**	182 (100.0)
**Inpatient mortality**	5 (2.7)

## Discussion

Prior to the current study, there were no published surveillance data specifically related to SPI in SA settings. Therefore, the findings of the current study are discussed in the context of published studies which did not differentiate between varying severities of intussusception. The profile of the study sample was in keeping with the findings of an earlier general surveillance study of paediatric intussusception in SA, in that the majority of patients in the study sample were <1 year old, male, and of black African ethnicity [3]. That general surveillance study also reported a much higher number of intussusception cases during the warmer seasons (spring and summer), which the current study appears to confirm [3]. The duration of hospitalization in the current study was 5.0 days. This is similar to the median duration of 6.0 days reported in another study of paediatric intussusception involving several low and middle-income countries [5]. All patients with SPI in the current study required surgical intervention. These patients were referred from lower-level healthcare facilities, where pneumatic reduction was attempted but had failed to resolve the condition. A large proportion of SA patients with paediatric intussusception present late to healthcare facilities [3]. Pneumatic reduction for intussusception is most effective if performed within two days following the emergence of symptoms [2], which explains the high rate of surgical intervention in SA reports on the condition [2,3]. The incidence of mortality in the current study was much lower than the 9.4% that was previously reported for intussusception in SA settings [3]. Access to quaternary-level healthcare resources, such as specialist paediatric surgeons and paediatric intensive care services, might explain the improved survival reported in the current study.

Although there were patients with SPI identified from ten out of the eleven district municipalities comprising KZN, the eThekwini and King Cetshwayo were the municipalities which had the highest proportion of patients with SPI in this study. These municipalities should be targeted for interventions which aim to address the burden of SPI in these settings. Interventions which address paediatric intussusception through “primordial”/primary and secondary prevention would be of most benefit in this situation. “Primordial” and primary prevention seek to address potential risk factors and risk behaviors for a disease in the wider community [6]. Hand washing campaigns in hospitals have been shown to reduce nosocomial rota-virus infection, an etiological agent of intussusception, within these facilities [7,8]. Similar campaigns which seek to improve the knowledge and awareness of appropriate hygiene practices in the general community might assist with reducing the occurrence of gastrointestinal infections which underlie paediatric intussusception in this population. Efforts to increase the uptake of vaccinations which protect against gastrointestinal infection and diarrhoeal disease [9,10], would also fall under the umbrella of primary prevention. Increasing access to potable water and sanitation would also contribute to reducing the risks of intussusception associated with gastrointestinal infection and diarrhoeal disease [10]. Regrettably, recent reports from the eThekwini municipality suggest a service backlog which might take up to 37 years to complete [11]. The early diagnosis of disease forms the basis of secondary prevention. Early diagnosis of disease is often coupled with early treatment, and this reduces the possibility of the disease advancing in severity [6].

Failures in secondary prevention are clearly illustrated with intussusception, where delayed diagnosis is associated with increased severity and more complex management of the condition [3]. Parents might delay taking their children to hospital, which in turn delays the diagnosis of intussusception. There are two possible reasons why parents might delay taking their children to a public hospital. Firstly, some parents might have insufficient knowledge and awareness of intestinal obstruction or intussusception [12] and therefore would not suspect that their child has a potentially life-threatening condition which requires urgent medical treatment. Secondly, parents might initially attempt to treat the condition over several days with home remedies or traditional methods [2,3]. It would appear that improving the knowledge and awareness of paediatric intussusception and/or intestinal obstruction amongst parents might reduce delays in presentation to a healthcare facility and subsequent diagnosis of the condition. Specific areas of knowledge and awareness which might require improvement are signs/symptoms of intussusception, intestinal obstruction, consequences if the condition is not diagnosed early, and the futility of home remedies in managing the condition. The most feasible way of increasing the knowledge and awareness of paediatric intussusception amongst parents would be to have information sharing and educational campaigns at clinics where these parents will bring their children in for compulsory vaccinations during the first year of life. This approach can be extended to the general community through the use of community health workers. From the healthcare practitioners´ point of view, paediatric intussusception can be a diagnostic challenge [2].

A large proportion of patients do not present with the “classic triad” of colicky abdominal pain, stool which is red and jelly-like, and a palpable abdominal mass [13]. Misdiagnosis on initial presentation is therefore a real possibility. Abdominal ultrasound has demonstrated excellent sensitivity and specificity for paediatric intussusception [14,15] and should be used in conjunction with the clinical examination to reduce the chances of misdiagnosis. The additional benefits of ultrasound include its widespread availability [16], its non-invasive approach [17] and that it can even be performed or interpreted by junior healthcare practitioners [15]. The current study was not without limitations. All data were obtained from patients attending a quaternary hospital in KZN, and the patient profile observed in this study might not necessarily apply to patients with SPI in other SA provinces. The electronic hospital admissions system from which the study data were obtained does not collect other relevant clinical information, such as signs/symptoms and risk factors, which could have been used to improve the description of the study sample. Recurrence, re-operation and other post-discharge outcomes were not investigated in this study. There is also a possibility that a small number of patients might have temporarily relocated to the eThekwini municipality for their surgery and might have provided this temporary address on admission to hospital.

## Conclusion

This study reports the geospatial distribution of SPI in KZN province, SA. Two KZN district municipalities, namely the eThekwini and King Cetshwayo municipalities, were identified for interventions aimed at combating SPI. Several preventative interventions (at the “primordial”, primary and secondary levels of prevention) have been proposed in this manuscript. The feasibility, uptake, and effectiveness of these recommended interventions should be investigated in the eThekwini and King Cetshwayo municipalities.

### What is known about this topic

- Cases of paediatric intussusception from SA are described as more severe when compared with cases from high-income countries;
- Many patients with SPI will be managed at quaternary healthcare facilities, which has important healthcare cost and resource utilization implications in a resource-limited setting such as SA.

### What this study adds

- Two district municipalities in KZN, SA were identified for primary and secondary prevention interventions targeting SPI;
- Potential examples of primary and secondary prevention interventions targeting SPI in this setting have also been proposed.
